# Psychological resources and flexibility predict resilient mental health trajectories during the French covid-19 lockdown

**DOI:** 10.1038/s41598-022-14572-5

**Published:** 2022-06-23

**Authors:** Nicolas Pellerin, Eric Raufaste, Maya Corman, Frederique Teissedre, Michael Dambrun

**Affiliations:** 1 CLLE, CNRS UMR 5263, Universite Toulouse 2 Jean Jaures (UT2J), 5 Allees A. Machado, 31058 Toulouse Cedex, France; 2 LAPSCO, CNRS UMR 6024, Universite Clermont Auvergne, Clermont-Ferrand, France

**Keywords:** Quality of life, Anxiety, Risk factors, Depression, Psychology and behaviour

## Abstract

The implementation of lockdowns and the Covid-19 pandemic situation have negatively impacted mental health (anxiety, depression). However, little is known about individual differences in the longitudinal reactions to lockdown. We designed a longitudinal study (a) to identify the various trajectories of symptoms of depression and anxiety in the general population during and after lockdown; (b) to determine which positive psychological resources prevent individuals from falling into groups with the most severe trajectories; (c) to test the mediating role of psychological flexibility. We collected and analysed longitudinal data on a sample of French participants (*N* = 1399, M_age_ = 43.4; SD_age_ = 12; 87.8% women) during the end of the first lockdown. Participants were asked to report their psychological resources and (in)flexibility at baseline and symptoms of anxiety and depression at each measurment occasion (five weekly observations from 17 March to 11 May 2020, including baseline). Using growth mixture modelling, seven dynamic profiles of symptoms were identified: four for depression and three for anxiety. Resilience emerged as the most frequent trajectory. Wisdom, optimism, hope, self-efficacy and peaceful disengagement significantly prevented individuals from belonging to the symptomatic groups. Moreover, psychological flexibility emerged as a significant mediator of these effects. This study highlights the importance of cultivating protective factors and psychological flexibility to prevent mental health damage during potentially traumatic events (PTE) and to favour resilience trajectories.

## Introduction

There is growing evidence that the Covid-19 pandemic situation degraded mental health in the general population^1^. Although lockdowns probably help reduce the circulation of the virus^2^, such measures are so extreme, in terms of restrictions of liberty and disruption of social interactions, that they can lead to further deterioration in mental health and well-being^3–7^. While previous studies have shown that people differ considerably in their responses to potentially traumatic events (PTEs) and that most people appear to be relatively resilient to them^8^, to date little is known about the heterogeneity of mental health responses to lockdown. Following a brief examination of the resilience construct, we consider the recent literature on psychological functioning during lockdown and pandemic situations which suggests the existence of individual differences in anxiety and depression trajectories. Among protective factors, we argue that the availability of psychological resources can explain individual differences in resilience. Finally, we propose that psychological flexibility may mediate the positive effects of psychological resources. We then report a longitudinal empirical investigation during and after the French lockdown to put these hypotheses to the test.

### Psychological resilience in the Covid-19 pandemic context

The study of resilience has received more and more attention, with very different theoretical and operational approaches^9^. Some conceptualise resilience as a dynamic process causing positive adaptation^10^ or as an individual disposition to bounce back from adversity^11^. In this paper, resilience is seen as the result of longitudinal psychological adjustment following PTEs. The maintenance of mental health and well-being during and after acute, short-term stressors has been termed ‘minimal-impact resilience’, as opposed to ‘emergent resilience’, which is long-term adaptation following persistent stressful contexts^12^. Because lockdown is intended to be temporary, resilience is understood here in the sense of minimal-impact resilience. We therefore adopted the definition of resilience proposed by Bonanno^13^ as ‘the ability of adults in otherwise normal circumstances who are exposed to an isolated and potentially highly disruptive event (…) to maintain relatively stable, healthy levels of psychological and physical functioning’ (p. 20).

Covid-19 has been shown to have a significant impact on mental health^1^. Studies have shown that people in lockdown were more likely to suffer from depression and anxiety than before the pandemic^14,15^. Quarantine, prolonged quarantine especially, showed adverse psychological effects, such as post-traumatic stress, confusion, anger, fear of infection, frustration, boredom, inadequate information, financial loss and stigma^16^. The first lockdown in France (from 17 March to 11 May 2020), as for many countries, combined many aggravating factors, such as a particularly long duration (1 month and 25 days) and negative public attitudes around its implementation i.e., perceived excessive media coverage of the pandemic, substantial economic damage and poor communication by the authorities^17^. In this respect, the pandemic and lockdown contexts undoubtedly represent a PTE that has a negative impact on mental health.

There is, however, a non-negligible barrier to the study of resilience during the first lockdown. Observing psychological reactions or non-reaction to PTEs requires longitudinal designs, i.e., multiple assessments^18^. Ideally, the measurement should be done at three different times: before, during and after the PTE^19^, so that the resilient outcome can be referenced to a baseline to observe the short-term and long-term impacts of PTEs. However, the implementation of the first lockdown was not predictable, preventing researchers from investigating individuals’ baseline mental health before lockdown. Studying a lockdown followed by unlockdown might serve as a relevant way to compare lockdown and not-lockdown situations. The moment of unlockdown itself might also be of particular interest and has been relatively understudied. People may differ in their mental reactions near the end of the lockdown. For example, unemployed people might experience decreased anxiety and depression in response to an actual or anticipated unlockdown. Conversely, teleworking employees might undergo increased anxiety as they approach the time to return to their workplace—particularly those who use public transportation where contamination is possible. We use the term ‘(un)lockdown’ throughout this document to refer to the entire process that begins during lockdown and continues just after unlockdown.

As already mentioned, longitudinal analyses are necessary to examine the developmental trend of psychopathological symptoms or resilience during (un)lockdown^18,19^. A recent review of available studies associated with the Covid-19 pandemic provides a good picture of the general trends of mental health trajectories^20^. For example, the trajectories of anxiety and depression of a large and representative sample of UK citizens progressively decreased for 20 weeks after the start of lockdown, with the steepest decrease during the period of strictest lockdown^21^. However, approaches used to date have only assessed the average population trajectory and failed to capture the heterogeneity of individuals’ mental health trajectories^22^. Pre-pandemic research on the impact of PTEs uncovered the existence of not one single but several distinct trajectories of anxiety and depression in the same population^23–26^. Such diversity might also hold in the pandemic and (un)lockdown contexts so that limiting the analysis to general population trends may mask substantive particularities. Bonnano’s team studied various contexts involving psychological adaptation to an aversive disruptive event such as the death of a spouse or the World Trade Center attack^27,28^. According to Bonanno, in the case of minimal-impact resilience, the main trajectories are *resilience* (characterised by little change in normal functioning over time), *recovery* (which implies a significant change in psychological functioning before a return to normal), *delayed response* (little change during the aversive event but the appearance of a delayed deleterious change in psychological functioning) and the state of *chronic disruption* (maintenance of a dysfunctional psychological state over the long term)^13^. A review of studies found that most individuals’ clinical trajectories after PTEs followed a pattern of resilience (65.7% across populations), followed by recovery (20.8%), chronic disruption (10.6%) and delayed responses (8.9%)^8^.

The present study aimed at evaluating group differences in anxiety and depression trajectories in the context of (un)lockdown. More generally, as observed in pre-Covid studies, we expected to find groups differing in substantive and persistent symptomatology. More precisely, we predicted that individuals would differ substantively in how they reacted to (un)lockdown in terms of depression and anxiety symptomatology, but that a significant proportion of individuals would be resilient.

### The positive psychological resources favoring resilience in terms of anxiety and depression

One of the main questions that resilience theory can be used to tackle is: why are some persons affected by (un)lockdown and the general pandemic context but not others? When comparing the resilient group with the others (i.e., individuals who report symptoms above the symptomatologic threshold at one point at least during the study period), the predictor variables can be considered risk factors or protective variables, depending on the direction of the relationship. Previous studies on resilience to PTEs have found many such factors: demographics, exposure, personality, social and economic resources, past and current stress, positive emotions, coping and appraisal, flexibility and genes^12,22^. In general, there are no predominant resilience factors against one particular PTE but rather multiple factors with relatively small effects^12^. For example, research on resilience in the face of natural disasters associated with climate change has found numerous factors, such as distal and proximal exposure to climate disasters, individual characteristics (demographics, biological vulnerability, personality coping and emotion regulation, social support), family and community^29^. Therefore, researchers were prompted to include many potentially relevant factors to find the best resilience profiles in the Covid-19 pandemic^30^.

With the development of positive psychology, there is growing evidence that positive mental dispositions or strength of character contribute to human fulfilment in a wide range of areas^31,32^. In particular, many positive psychology constructs have proven to be effective in improving well-being and relieving the symptoms of depression and anxiety^33–35^. Many studies have already identified some of these protective psychological factors, such as mindfulness, perceived social support^36^, optimism, resilience (conceptualized as an individual disposition^37^), good stress response recovery and positive appraisal^38^. However, these studies did not operationalise mental health longitudinally. Another limitation is that the authors limited their scope to a few selected psycho-social resources. A longitudinal study conducted during the French lockdown of the Covid-19 pandemic uncovered a set of psychological resources that increased various forms of happiness^39^. The notion of psychological resources refers to positive mental dispositions that contribute to personal well-being and resilience. Based on three criteria (the relation of the resource with well-being had to be theoretically and empirically established and a psychometrically sound instrument had to be available for the resource), nine well-studied psychological resources were selected: self-efficacy^40^; optimism^41^; hope^42^; personal wisdom^43^; self-transcendence^44^; gratitude toward the world^45^; gratitude of being^46^; peaceful disengagement^46^; and acceptance^47^. Results showed that most of these constructs had protective effects on well-being, enhancing happiness averages and reducing the lockdown’s negative impact and perceived economic and health threats^39^. Interestingly, various happiness dimensions (emotional, psychological, social and inner well-being) had their own set of protecting variables. Therefore, it can be expected that positive psychological resources also differently influence depression and anxiety trajectories during and after PTEs.

In this study, we sought to identify psychological resources that are protective against the symptomatologic trajectories of depression and anxiety in the context of (un)lockdown and the Covid-19 pandemic. Most of the resources mentioned above were selected for this purpose. Overall, we examined the protective role of psychological resources by testing their ability to predict a trajectory of resilience compared to a symptomatologic trajectory in terms of anxiety or depression. Specifically, we predicted that most of the psychological resources previously identified would increase the likelihood of a resilience trajectory and decrease the likelihood of a trajectory symptomatic of anxiety or depression.

### Psychological flexibility as a mediating process

The last purpose of this study was to test a mechanism that would explain the expected protective role of psychological resources. Because psychological flexibility is centrally related to depression and anxiety outcomes, it might explain the effects of psychological resources on resilience. Psychological flexibility is a broad concept that reflects a person’s ability to easily adapt their cognition and behaviour according to their needs^48^. Part of psychological flexibility is regulatory (or coping) flexibility—that is, the ability to adapt one’s behaviour in response to the demands of the environment^49^. This construct has been identified as a key to emerging resilience against PTEs in general^49^ and also potentially in the Covid-19 crisis and lockdowns in particular^30^. The construct of psychological flexibility is also embedded in the Acceptance and Commitment Therapy (ACT) principles, within the third wave of Cognitive and Behavioral Therapy, although it differs in certain respects from regulatory flexibility. We propose that the two approaches are not contradictory but rather complementary in explaining how and why people respond differently to PTEs.

The core principle of ACT is that negative experiences such as stress, loss or pain are inevitable. People engaged in fighting against these aversive experiences tend to reinforce their negative aspects, which keeps them away from valued and meaningful action^50^. In ACT, psychological flexibility is viewed as the ability to stay in contact with the present moment, noticing and accepting the way feelings, bodily sensations, and thoughts occur while doing what matters in agreement with one’s own values^50^. Psychological inflexibility is characterised by rigid cognitive, emotional and behavioural functioning, in which individuals facing stressful situations react and act while trying to control and avoid internal and external negative experiences (bodily sensations, thoughts, feelings)^51^. This approach seems particularly relevant to have a better understanding of the psychological processes that develop and maintain psychological distress in a pandemic context^52,53^ and to propose effective interventions for people^52^. Indeed, psychological flexibility has been identified as a transdiagnostic process that protects against the emergence and maintenance of several emotional disorders such as depression, anxiety and suicidal ideation^54,55^. Psychological flexibility and inflexibility are considered as close but distinct processes that are not just two extremes on the same continuum^56^. Psychological flexibility is related to better mental health, self-compassion and well-being^57,58^. Some studies have even approached psychological flexibility as a resilience factor for individuals with post-traumatic stress disorder, depression or chronic pain^54,59^. More recently, and directly related to the pandemic situation, McCracken et al.^60^ showed that psychological flexibility was negatively related to anxiety and depression.

Some psychological resources promoting resilience, such as optimism, hope and self-efficacy, are related to psychological flexibility^61^. These relationships, and the fact that psychological flexibility has been robustly associated with mental health, has prompted researchers to test for the mediating effects of this construct. Psychological flexibility was found to be a mediator between the fear of negative evaluation and psychological vulnerability^62^ or between childhood aversive events and mental health outcomes^63^. Recent studies pertaining to the Covid-19 pandemic have taken a similar approach. Psychological (in)flexibility was found to mediate the relationship between coronavirus stress and psychological health and well-being, along with some other psychological resources^64,65^. Moreover, they found that psychological inflexibility mediates the relationship between optimism–pessimism and psychological problems in adults (i.e., depression, anxiety, somatisation). Wąsowicz et al.^66^ identified psychological flexibility as a mediating factor between negative emotions and general well-being. Therefore, we suggest that psychological flexibility is one of the best predictors of a resilient trajectory and that the protective effect of the other psychological resources on resilience is mediated, at least partly, by psychological flexibility.

### Summary of the study hypotheses

In summary, we sought to test three hypotheses:

#### **H1**

Individuals differ substantively in how they longitudinally react to (un)lockdown in terms of depression and anxiety symptomatology, with significant proportion of individuals displaying a resilient trajectory.

#### **H2**

Psychological resources predict resilient trajectories of anxiety and depression against symptomatological trajectories.

#### **H3**

Psychological flexibility mediates the protective effects of psychological resources.

## Methods

### Participants and procedure

All the procedures performed in these studies were reviewed and approved by the ethics committee of Toulouse University (Comité d’Ethique de la Recherche (CER) à l’Université Fédérale de Toulouse). The study was performed in accordance with the Helsinki Declaration. The participants gave their informed consent to participate in this study.

We recruited volunteers via an advertisement on social networks in France during the beginning of the first lockdown. A total of 1399 participants fully completed the first questionnaire and provided their email addresses (wave 1). This questionnaire included questions about individual characteristics and contexts and measures of psychological resources, psychological flexibility, and depression and anxiety indexes. Then, each week for four weeks, participants were prompted to respond to a shorter questionnaire that included the depression and anxiety indexes (waves 2 to 5). Table 1 presents the demographics of the participants for each wave.

**Table 1:** Sociodemographic characteristics of participants in each wave.

Wave	1	2	3	4	5
Start date	28-04	06-05	13-05	20-05	27-05
Lockdown	Yes	Yes	No	No	No
Total $\mathrm{N}$	1399	924	849	688	661
**Gender**					
Men	171 (12.2%)	110 (11.9%)	97 (11.4%)	76 (11%)	72 (10.9%)
Women	1228 (87.8%)	814 (88.1%)	752 (88.6%)	612 (89%)	589 (89.1%)
**Age **$\mathbf{M}$** (SD)**	43.4 (12.0)	43.5 (11.7)	44.0 (11.8)	44.4 (11.9)	44.5 (12.1)
18–25	112 (8%)	63 (6.8%)	53 (6.2%)	42 (6.1%)	42 (6.4%)
25–35	261 (18.7%)	178 (19.3%)	154 (18.1%)	123 (17.9%)	120 (18.2%)
35–45	445 (31.8%)	296 (32%)	275 (32.4%)	211 (30.7%)	195 (29.5%)
45–55	338 (24.2%)	233 (25.2%)	217 (25.6%)	183 (26.6%)	173 (26.2%)
55–65	189 (13.5%)	122 (13.2%)	117 (13.8%)	98 (14.2%)	99 (15%)
65–78	54 (3.9%)	32 (3.5%)	33 (3.9%)	31 (4.5%)	32 (4.8%)
**Work**					
Newly inactive	342 (24.4%)	250 (27.1%)	229 (27%)	191 (27.8%)	183 (27.7%)
Full teleworking	358 (25.6%)	237 (25.6%)	208 (24.5%)	159 (23.1%)	154 (23.3%)
Partial teleworking	120 (8.6%)	80 (8.7%)	84 (9.9%)	68 (9.9%)	59 (8.9%)
Working outside home	196 (14%)	126 (13.6%)	114 (13.4%)	93 (13.5%)	86 (13%)
Inactive	383 (27.4%)	231 (25%)	214 (25.2%)	177 (25.7%)	179 (27.1%)

Wave 1 corresponds to the initial measurement time; waves 2 to 5 are weekly follow-ups. Each participant responded to at least two waves (including wave 1).

### Materials

#### Depression and anxiety

Both depression and anxiety were assessed using the Hospital Anxiety Depression Scale (HADS)^67,68^. The HADS comprises seven items using a Likert scale that capture the frequency of experiences from 0 (‘never’) to 3 (‘almost always’). Summing anxiety and depression items gives scores that can serve to identify depression and anxiety symptomatology^67^. Scores of 7 or less indicate no symptomatology, scores of 8 to 10 signal doubtful cases, and scores of 11 and above denote definite symptomatic cases. Cronbach alphas were satisfactory for depression ($\alpha$ = 0.83) and anxiety ($\alpha$ = 0.79).

#### Psychological resources

All psychological resources were measured at the first wave using Likert scales ranging from 1 = ‘strongly disagree’ to 7 = ‘strongly agree’.

##### Hope, optimism, and self-efficacy

We assessed hope (e.g., ‘If I should find myself in a jam, I could think of many ways to get out of it’), optimism (e.g., ‘I am looking forward to the life ahead of me’), and self-efficacy (e.g., ‘I am confident that I could deal efficiently with unexpected events’) using the Compound-Psychological-Capital questionnaire (CPC-12)^39,69^. Reliabilities were satisfactory for hope ($\alpha$ = 0.75), optimism ($\alpha$ = 0.79), and self-efficacy ($\alpha$ = 0.82).

##### Personal wisdom

Personal wisdom was assessed with the 12-Item Abbreviated Three-Dimensional Wisdom Scale (3D-WS-12)^39,70^, which uses four items to measure each of three dimensions of wisdom, as theorized by Ardelt^71^: cognitive (e.g., ‘A problem has little attraction for me if I don’t think it has a solution.’), affective (e.g., ‘Sometimes I feel a real compassion for everyone’), and reflective (e.g., ‘When I am confused by a problem, one of the first things I do is survey the situation and consider all the relevant pieces of information’). The personal wisdom measure was marginally reliable ($\alpha$ = 0.64).

##### Self-transcendent wisdom

Self-transcendent wisdom was assessed using the most recently published version of the Adult Self-Transcendence Inventory (ASTI)^39,72^. The classical version of the ASTI measured self-transcendence as a single dimension^44^. Koller et al.^72^ used a mixed-method procedure to assess the ASTI dimensionality, including item evaluations by wisdom and psychometric experts and quantitative analysis using item response theory. They found five non-overlapping dimensions: ‘self-knowledge and integration,’ ‘peace of mind,’ ‘non-attachment,’ ‘self-transcendence,’ and ‘presence in the here-and-now and growth.’ We used all seven items of the self-transcendence dimension as a measure of self-transcendent wisdom (e.g., ‘I feel that my individual life is part of a greater whole.’, $\alpha$ = 0.77).

##### Gratitude toward the world

The 6-item Gratitude Questionnaire (GQ-6)^39,45^ was used to assess dispositional gratitude (e.g., ‘I have so much in life to be thankful for,’ or ‘I am grateful to a wide variety of people’). This measure had adequate reliability in our sample ($\alpha$ = 0.78).

##### Gratitude for being and peaceful disengagement

We used the Minimalist Well-Being Scale to assess gratitude for being and peaceful disengagement^46,73^. Four items captured the disposition to be grateful for just being (e.g., ‘I feel grateful that I am alive’), and seven items captured peaceful disengagement (e.g., ‘It feels good to do nothing and relax’). Both construct reliabilities were satisfactory (gratitude for being: $\alpha$ = 0.83; peaceful disengagement: $\alpha$ = 0.78).

#### Psychological flexibility

We used two measures of psychological flexibility.

- Psychological flexibility was assessed with the Acceptance and Action Questionnaire II (AAQII)^51,74^. This seven-point Likert scale (from 1 = ‘never true’ to 7 = ‘always true’) measures acceptance and action, two core constructs of psychological flexibility, with a score totaled over 10 items (e.g., ‘My thoughts and feelings do not get in the way of how I want to live my life’). Participants with higher total scores experience higher levels of psychological flexibility ($\alpha$ = 0.87).
- Psychological inflexibility was assessed with the Avoidance and Fusion Questionnaire for adults (AFQ)^75,76^. This four-point Likert scale (from 0 = ‘not true at all’ to 4 = ‘very true’) comprises items measuring the degree of adherence to 17 statements (e.g., ‘I can't stand feeling pain or hurt in my body’) reflecting experiential avoidance and cognitive fusion. The 17 items were aggregated to provide a total score ($\alpha$ = 0.87). Higher scores correspond to higher levels of psychological inflexibility.

#### Control variables: lockdown context

Participants reported the quality of their lockdown situation regarding the quality of their environment, relationships, and solitude. The quality of the physical environment in which they lived during the lockdown was assessed using a 5-point Likert scale (1 = ‘extremely bad’, 5 = ‘extremely good’). Solitude was assessed by asking whether the participant was alone in his home during the lockdown. Individuals who reported not being alone in their homes rated the quality of their relationships with the people with whom they were locked in using a 5-point Likert scale (1 = ‘extremely bad,’ 5 = ‘extremely good’). People alone in lockdown were not presented with this question and the missing value was replaced by the neutral response (‘neither good nor bad’). This replacement was done in order to add this variable in the models while not removing all responses from people who are alone in their home. Note that the effect of this variable does not change in alternative models where all responses of lonely people are removed.

### Data analysis

R^77^ was used for all analyses. The data and R scripts of the analyses are available online in an open directory (https://osf.io/q2e6h/).

To identify distinct groups in trajectories of depression and anxiety, we performed growth mixture modelling with the *hlme* function of the *lcmm* package^78^. Growth mixture modelling (GMM) can be seen as a combination of latent class growth and linear mixed analyses^79,80^. Latent class growth analysis (LCGA) relies on the latent growth curve modelling approach in which repeated variables are used to estimate latent intercepts (i.e., the level on the variable at the first time point) and slopes (i.e., the rate of change in the outcome over time) for each individual. Then, individuals with similar intercept and slope parameters are classified into subgroups. LCGA is a particular case of GMM in which no within-group variance of the parameters is allowed. In other words, all individuals’ growth trajectories are assumed to be that of the group, but it is unlikely that this assumption holds true with empirical data. GMM is more flexible as it enables specific intercept and slope parameters to be random, in the same manner as the linear mixed modelling framework.

Time in the GMM was treated as the true time since the first measurement in weeks. In this way, missing measurement occasions and random time intervals between two measurement occasions do not bias the final results. Because we expected trajectories to change during the (un)lockdown process for some classes, a quadratic slope was estimated along with the intercept and the linear slope. We used the following steps to select the best number of latent classes. (1) We computed 1 to 7 classes for a set of models with increasingly complex random structures: no random structure; random intercept only; random intercept and linear slope; and random intercept, linear and quadratic slopes. (2) Using the Bayesian information criterion (BIC), the first six models on all 28 possibilities with the best fit were selected and plotted along with their fit indices for comparison. (3) Finally, the best model was selected following these criteria: (a) best fit in terms of BIC; (b) classes not too small (i.e., each class had to represent at least 5% of the total sample); (c) the classes had to represent meaningful trajectories and each new complexity level had to provide relevant new information. Three authors (the first, second and last) deliberated and decided on the best model based on these criteria.

To test H2, class membership was predicted by fitting multinomial logistic regression models using the *multinom* function of the *nnet* package^81^ in a two-step process. The multinomial logistic regression models provided odds ratios that indicate the probabilities of being assigned to each class rather than the reference class. We first calculated zero-ordered models for each independent variable as unique predictors of class membership. Only variables that proved to be a significant predictor for at least one class membership in the zero-ordered models were entered in the next models. In the first step, all psychological resources, except psychological flexibility, and control variables such as age and sex were simultaneously entered. In the second step, psychological inflexibility and flexibility were added. A significant effect of a variable in steps 1 and 2 is not reported in the text of the Result section if the corresponding zero-order effect was either (1) non-significant or (2) in the opposite direction. Variance inflation factors (VIF) were calculated with the *car* package’s *vif* function^82^.

Lastly, we tested H3 using the method of Iacobucci^83^, which provides general guidance for the mediation analysis with categorical variables. The method consists in calculating the indirect path (‘ab path’) using estimates and standard errors of the effect of the independent variable on the mediator (‘a path’) and of the mediator on the dependent variable (‘b path’). In our data, the *a* path parameters were inferred from a linear regression model with psychological flexibility as DV and all psychological resources entered as predictors. The *b* path parameters came from step 2 of the multinomial logistic regression described above. We only tested the mediation model for a combination of IV, mediator, and DV when both the total effect of the IV on the DV (i.e., the effect of the psychological resource on class membership in step 1) and the *b* path (i.e., the effect of psychological flexibility on class membership on step 2) were significant. When one of these paths was not significant, we assumed that the mediation was not significant^84^.

## Results

Means, standard deviations, VIF and correlations of the study variables are presented in Table 2. All VIF were below the cutoff of 3, indicating that multicollinearity was not an issue^85^.

**Table 2:** Descriptive statistics and correlation matrix of the study variables as measured in wave 1.

	$M$	$SD$	VIF	1	2	3	4	5	6	7	8	9	10	11
1. Depression	10.01	4.17		–										
2. Anxiety	17.54	3.96		0.59	–									
3. Psychological flexibility (AAQ-II)	4.42	0.97	2.71	− 0.60	− 0.57	–								
4. Psychological inflexibility (AFQ)	1.82	0.73	2.31	0.55	0.50	− 0.73	–							
5. Gratitude-world (GQ-6)	4.99	1.01	1.93	− 0.28	− 0.41	0.39	− 0.32	–						
6. Self-Transcendence (ASTI)	4.91	1.01	1.39	− 0.14	− 0.19	0.18	− 0.13	0.48	–					
7. Wisdom (3D-WS)	4.62	0.71	1.89	− 0.38	− 0.42	0.57	− 0.56	0.41	0.29	–				
8. Optimism (CPC-12)	4.72	1.25	1.81	− 0.33	− 0.44	0.41	− 0.30	0.47	0.26	0.29	–			
9. Self-efficacy (CPC-12)	5.32	1.08	1.82	− 0.39	− 0.45	0.49	− 0.38	0.34	0.26	0.49	0.44	–		
10. Hope (CPC-12)	5.00	1.05	2.15	− 0.42	− 0.53	0.55	− 0.44	0.46	0.27	0.44	0.58	0.58	–	
11. Gratitude-being (MW-BS)	5.23	1.06	1.68	− 0.22	− 0.39	0.37	− 0.24	0.54	0.28	0.26	0.51	0.28	0.42	–
12. Peaceful disengagement (MW-BS)	5.18	0.96	1.4	− 0.45	− 0.45	0.36	− 0.28	0.31	0.30	0.20	0.34	0.40	0.41	0.34

All correlations are significant at $p$ < 0.001.

$M$ mean, $SD$ standard deviation, *VIF* variance inflation factor.

### Analyses of trajectories

#### Depression trajectories

Table 3 presents the six best models (BIC, parsimony indices, entropy and class distribution). The trajectories of all classes for the six models are provided in the supplemental materials (Fig. S1). The four best-fitting models of depression had random intercepts only, with numbers of classes ranging from 4 to 7. Three out of the six classes of the best model had sample sizes below the 5% cutoff. The second best-fitting model had four classes similar to those in model 1, but two small groups. Only one small group (*N* = 21, 1.5%) was still present in the 4-class model. This small group was systematically present in the four best-fitting models and described a meaningful and relevant trajectory of depression (depression levels increase linearly with time). Although the third and fourth best-fitting models showed parsimony indices very similar to that of the second, they had two or more very small additional clusters. Their additional clusters did not bring substantive new information over the second best-fitting model, which also had the best entropy value (i.e., 77). Therefore, the second best-fitting model, with four classes and random intercept, was identified as the best solution.

**Table 3:** Fit indices and class distributions of the six best growth mixture models for depression.

Rank	Random	${N}_{C}$	AIC	BIC	SABIC	Entropy	%.C1	%.C2	%.C3	%.C4	%.C5	%.C6	%.C7
**Depression**													
1	I	6	21,662.86	21,793.95	21,714.53	0.75	9.4	1.1	2.8	77.4	5.4	3.9	
2	I	4	21,736.78	21,825.92	21,771.91	0.77	1.5	7.9	79.6	11.1			
3	I	7	21,675.50	21,827.56	21,735.44	0.75	3.6	2.6	1.0	69.8	11.4	0.0	11.7
4	ISQ	7	21,661.97	21,840.25	21,732.25	0.52	1.0	2.5	16.7	9.4	0.0	66.6	3.7
5	I	5	21,763.66	21,873.78	21,807.07	0.75	78.9	4.6	2.6	2.7	11.2		
6	ISQ	3	21,779.88	21,874.26	21,817.08	0.76	81.7	7.9	10.4				
**Anxiety**													
1	IS	3	22,587.22	22,665.87	22,618.22	0.52	13.2	59.0	27.8				
2	IS	4	22,569.39	22,669.01	22,608.66	0.61	56.3	3.4	32.9	7.4			
3	I	6	22,543.54	22,674.63	22,595.21	0.53	37.0	31.4	3.2	17.5	6.8	4.1	
4	I	5	22,565.88	22,675.99	22,609.28	0.57	26.2	4.6	58.0	7.4	3.8		
5	IS	5	22,562.23	22,682.83	22,609.77	0.61	56.6	2.1	30.5	2.4	8.4		
6	ISQ	3	22,591.20	22,685.59	22,628.41	0.53	13.2	59.0	27.8				

The models are sorted by increasing BIC. The ‘Random’ column indicates the combination of random parameters in the model (‘I’ stands for intercept, ‘S’ for linear slope, and ‘Q’ for quadratic slope). The ${N}_{C}$ column gives the number of classes estimated in the model.

**Figure 1:**
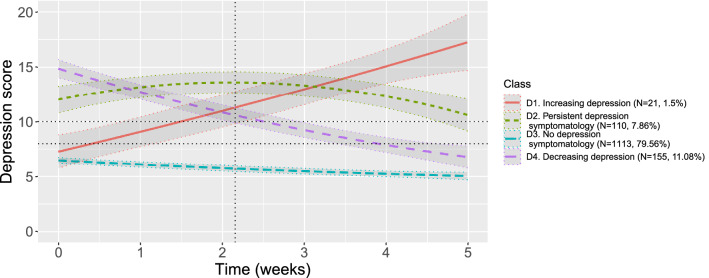
Predicted trajectories from selected growth mixture model of depression. Dashed horizontal lines represent symptomatic thresholds for depression (i.e., 8 and 10). The dashed vertical line depicts the moment unlockdown happened.

Figure 1 presents the trajectories of the selected model. Table 4 provides the fixed parameter estimates for each class. The four clusters identified are described as follows. Cluster D1, ‘increasing depression’ (*N* = 21, 1.5%), characterises people with initially asymptomatic levels of depression (though close to 8) and a depressive tendency that steadily increase, reaching the symptomatic threshold before the date of unlockdown and never ceasing to worsen until the end of data collection. Individuals in cluster D2, referred to as ‘persistent depression symptomatology’ (*N* = 110, 7.9%), have levels of depression above the symptomatic threshold in all waves with a slight initial increase until release from lockdown and a relative recovery, close to the symptomatic level in wave 5. This group represents people who experienced unlockdown negatively and were not relieved by it. Cluster D3, ‘no depression symptomatology’ (*N* = 1113, 79.6%), includes people with non-symptomatic levels of depression throughout the whole data collection period. This group is also characterised by a slight decrease in depression levels over time. Finally, cluster D4, ‘decreasing depression’ (*N* = 155, 11.1%), includes individuals who initially exhibited severe depression but whose symptoms progressively disappeared, and who reached non-symptomatic levels of depression in wave 5.

**Table 4:** Intercepts, linear and quadratic time parameters for each class of the selected growth mixture models.

	Parameter	$b$	SE	Wald	$p$
**Depression class**					
D1	Intercept	10.9	0.65	16.79	0
	Linear time	191.97	17.83	10.77	0
	Quadratic time	5.64	18.48	0.3	0.76
D2	Intercept	12.84	0.49	26.43	0
	Linear time	− 2.72	11.23	− 0.24	0.81
	Quadratic time	− 41.93	9.73	− 4.31	0
D3	Intercept	5.84	0.12	48.33	0
	Linear time	− 29.61	2.52	− 11.75	0
	Quadratic time	2.6	2.41	1.08	0.28
D4	Intercept	11.29	0.32	35.35	0
	Linear time	− 166.98	8.77	− 19.05	0
	Quadratic time	15.07	7.56	1.99	0.05
**Anxiety class**					
A1	Intercept	9.19	0.31	29.29	0
	Linear time	49.35	14.44	3.42	0
	Quadratic time	− 91.11	11.16	− 8.17	0
A2	Intercept	5.36	0.19	27.49	0
	Linear time	− 5.88	5.2	− 1.13	0.26
	Quadratic time	− 3.55	3.73	− 0.95	0.34
A3	Intercept	11.67	0.26	44.72	0
	Linear time	− 47.19	9.71	− 4.86	0
	Quadratic time	− 16.04	6.35	− 2.52	0.01

Time is coded in weeks.

#### Anxiety trajectories

Table 3 shows the parsimony indices and class distributions of the six best-fitting growth mixture models of anxiety. Class trajectories of these models can be found in the supplemental materials (Fig. S2). The first three best-fitting models had random intercepts and linear slope parameters for three to five clusters. The best-fitting model had three clusters of adequate size (13.2%, 59.0%, 27.8%, respectively). The second model had four classes, three classes identical to the first model and one additional weakly populated class (3.1%). More generally, models 2–6 presented at least one small class. Although not fitting at best in terms of AIC and SABIC, we decided to retain the first model, with three classes, random intercept and slope, as it provides well distributed classes and few categories.

**Figure 2:**
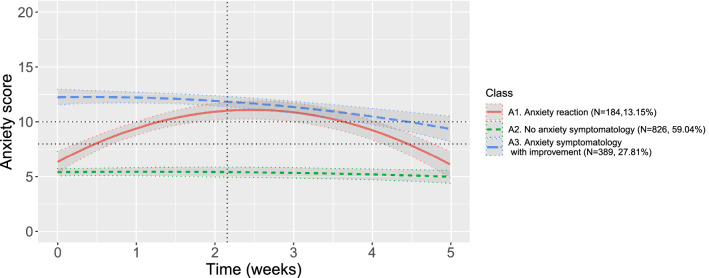
Predicted trajectories from the selected growth mixture model of anxiety. Dashed horizontal lines represent symptomatic thresholds for anxiety (i.e., 8 and 10). The dashed vertical line depicts the moment of unlockdown.

Figure 2 gives the predicted trajectories from the selected model. Table 4 presents the fixed parameter estimates for each class. The A1 cluster, ‘anxiety reaction’ (*N* = 184, 13.2%), represents people who started below the symptomatic threshold in anxiety, increased to symptomatic levels around unlockdown, and came back to their initial level two to three weeks later. Cluster A2, ‘no anxiety symptomatology’ (*N* = 826, 59.0%), includes people who presented no anxiety problems from the beginning to the end of the assessments. Cluster A3, named ‘anxiety symptomatology with improvement’ (*N* = 389, 27.8%), characterised people who started with high levels of anxiety—beyond the symptomatic threshold—and saw their anxiety slowly decrease, particularly after the end of lockdown.

### Multinomial logistic regressions

#### Predicting depression trajectories

Table 5 presents the odds ratios from the multinomial logistic models of depression trajectories for classes D1, D2 and D4 with the no depression symptomatology class (D3) as the reference group. All variables appeared to significantly predict at least one class membership in the zero-ordered models. Therefore, all variables were included in the subsequent models. Note that we do not report or discuss any significant effect on a particular class for a variable that was not significant or in the opposite direction in the respective zero-ordered model. The two steps in the table depict the two nested models with psychological resources and demographic variables in step 1 and adding psychological (in)flexibility in step 2. Odds ratios > 1 indicate that increases in the predictor variable make it likelier to be in the target class than in the reference class. In other words, a significant odds ratio < 1 indicates a protective effect of the predictor against belonging to the corresponding symptomatologic class. In contrast, a significant odds ratio > 1 indicates an adverse effect of that variable, implying a greater probability of belonging to the corresponding symptomatologic class. We only tested whether psychological flexibility would mediate the effects of a specific psychological resource on class membership when the effect of the psychological resource on psychological flexibility in step 1 was significant.

**Table 5:** Odds ratios of being in the depression symptomatology classes (Classes D1, D2, and D4) versus being in the no depression symptomatology class (D3) depending on psychological resources (Step 1) and psychological flexibility (Step 2), controlling for sociodemographic variables.

Predictor	Class D1 (N = 21) Increasing depression			Class D2 (N = 110) Persistent depression			Class D4 (N = 155) Decreasing depression		
	Z-O	Step 1	Step 2	Z-O	Step 1	Step 2	Z-O	Step 1	Step 2
Gratitude-world	1.03	1.06	1.08	0.73**	1.00	1.08	0.56***	0.99	1.09
Self-transcendence	1.21	1.31	1.24	0.98	1.34*	1.18	0.73***	1.15	1.02
Wisdom	0.95	0.93	1.35	0.47***	0.50***	1.03	0.26***	0.40***	0.89
Optimism	0.70*	0.60*	0.61*	0.69***	0.79*	0.83	0.53***	0.75**	0.77*
Self-efficacy	0.98	1.19	1.23	0.73**	1.16	1.22	0.41***	0.79*	0.86
Hope	0.80	0.84	0.97	0.64***	0.93	1.14	0.39***	0.68**	0.89
Gratitude-being	1.02	1.24	1.34	0.72***	0.98	1.14	0.67***	1.29*	1.41**
Peaceful disengagement	1.01	1.01	1.10	0.60***	0.65***	0.75*	0.40***	0.54***	0.60***
Psychological flexibility	0.58*		0.46	0.25***		0.27***	0.17***		0.33***
Psychological inflexibility	1.61		1.01	3.79***		1.31	7.41***		2.05**
Age	0.99	0.98	0.99	0.98*	0.98	0.99	0.97***	0.98*	0.99
Gender-female	0.67	0.57	0.45	2.02	1.84	1.42	3.34**	3.03*	2.39
Work-newly inactive	1.07	1.09	1.07	0.91	0.82	0.85	0.91	0.73	0.76
Work-partial teleworking	0.38	0.46	0.42	0.57	0.59	0.50	0.94	1.24	1.09
Work-working outside home	0.00	0.00***	0.00***	0.56	0.56	0.62	1.17	1.13	1.18
Work-inactive	0.61	0.66	0.61	0.70	0.69	0.64	1.00	0.93	0.91
Environment quality	1.00	1.10	1.14	0.78*	0.87	0.96	0.70***	0.79*	0.87
Relation quality	1.25	1.29	1.29	0.61***	0.67**	0.66**	0.68***	0.80	0.75*
Solitude	1.01	1.22	1.16	0.95	0.72	0.67	0.60*	0.45**	0.43**

Values in Step 1 and 2 columns are the odds ratios from the multinomial regression models predicting membership of the depression classes against no depression symptomatology class as the reference group (Class D3). The Z-O column gives the variable zero-ordered effects, with other variables not included in the model.

*p* < 0.05, *p* < 0.01, *p* < 0.001.

Let us consider the effects of the psychological resources for each of the symptomatologic clusters. Optimism appeared to be a protective variable in reducing the probability of belonging to the group of increasing depression (class D1) in step 1 and 2. Psychological flexibility did not appear as a significant protective variable in step 2 (it was marginally significant) and did not significantly mediate the effects of optimism (*z* =  − 1.29; *p* = 0.1). Note that as this class included very few people (*N* = 21), these results should be interpreted with caution.

In step 1, people were less likely to belong to the group with persistent depression symptomatology (class D2) with better initial scores of wisdom, optimism and peaceful disengagement. In step 2, wisdom and optimism effects were no longer significant, and psychological flexibility showed very strong protective effects. Mediation analysis revealed that psychological flexibility mediated class membership for wisdom (*z* =  − 6.15; *p* < 0.001), optimism (*z* = − 1.84; *p* < 0.05) and peaceful disengagement (*z* = − 4.35; *p* < 0.001).

Finally, membership of the decreasing depression group (class D4) in step 1 was negatively predicted by wisdom, optimism, self-efficacy, hope and peaceful disengagement. Only psychological flexibility, optimism and peaceful disengagement were significant protective variables in step 2. Again, the effects of wisdom (*z* = − 5.44; *p* < 0.001), optimism (*z* = − 1.81; *p* < 0.05), self-efficacy (*z* = − 2.91; *p* < 0.01), hope (*z* = − 4.66; *p* < 0.001) and peaceful disengagement (*z* = − 4.35; *p* < 0.001) were significantly mediated by psychological flexibility. In contrast, initial levels of psychological inflexibility and gratitude of being increased the probability of being part of that group (but zero-ordered effect of gratitude of being was in the opposite direction). Psychological inflexibility appeared as a mediator for the effects of wisdom (*z* = − 2.99; *p* < 0.01), hope (*z* = − 2.64; *p* < 0.01) and peaceful disengagement (*z* = − 2.59; *p* < 0.01) but not for optimism (*z* = − 0.84; *p* = 0.2) and self-efficacy (*z* = − 0.3; *p* = 0.38).

#### Predicting anxiety trajectories

Table 6 presents the odds ratios from the two stepped multinomial logistic models with no anxiety problem as the reference group. All variables appeared to significantly predict at least one class membership in the zero-ordered models and were thus included in steps 1 and 2.

**Table 6:** Odds ratio of being in the anxiety symptomatology classes (Classes A1 and A3) versus being in the no anxiety symptomatology class (A2) depending on psychological resources (Step 1) and psychological flexibility (Step 2), controlling for sociodemographic variables.

Predictor	Class A1 (N = 169) Anxiety reaction			Class A3 (N = 317) Anxiety with improvement		
	Z-O	Step 1	Step 2	Z-O	Step 1	Step 2
Gratitude-world	0.94	1.17	1.20	0.43***	0.80*	0.84
Self-transcendence	1.16	1.43***	1.38**	0.73***	1.40***	1.28*
Wisdom	0.58***	0.54***	0.68*	0.27***	0.44***	0.73*
Optimism	0.72***	0.77**	0.78**	0.45***	0.78**	0.82*
Self-efficacy	0.80**	1.13	1.14	0.39***	0.83	0.84
Hope	0.67***	0.81	0.87	0.27***	0.56***	0.64***
Gratitude-being	0.79**	0.88	0.90	0.47***	0.85	0.87
Peaceful disengagement	0.77**	0.81*	0.85	0.37***	0.53***	0.58***
Psychological flexibility	0.51***		0.67**	0.22***		0.59***
Psychological inflexibility	1.91***		1.07	5.02***		1.68**
Age	0.99	1.00	1.00	0.98**	0.99	1.00
Gender-female	2.39**	1.92*	1.66	1.41	1.09	0.84
Work-newly inactive	0.72	0.70	0.69	1.04	0.75	0.75
Work-partial teleworking	0.72	0.75	0.72	0.78	0.85	0.81
Work-working outside home	0.43**	0.47*	0.47*	0.73	0.58*	0.59
Work-inactive	0.61*	0.59*	0.57*	1.15	0.92	0.87
Environment quality	0.86	0.82	0.83	0.59***	0.69***	0.72***
Relation quality	0.88	0.87	0.88	0.57***	0.71***	0.70**
Solitude	0.71	0.55*	0.54*	1.15	0.67	0.66

Values in Step 1 and 2 columns are the odds ratio from the logistic regression models predicting membership of the anxiety classes with no anxiety symptomatology class as the reference group (Class A2). The column Z-0 depicts the zero-ordered effects of the variable, with other variables not included in the model.

*p* < 0.05, *p* < 0.01, *p* < 0.001.

Concerning membership of the anxiety reaction group (class A1), wisdom, optimism and peaceful disengagement appeared as protective variables in step 1. In step 2, wisdom effects decreased but was still significant, and optimism and peaceful disengagement effects did not change much. Psychological flexibility appeared to be protective and to significantly mediate the effects of wisdom (*z* = − 2.7; *p* < 0.01) and peaceful disengagement (*z* = − 2.45; *p* < 0.01) in the expected direction, and marginally for optimism (*z* = − 1.52; *p* = 0.06). Psychological inflexibility was not related to membership in this class.

Participants were less likely to be in the anxiety symptomatology with improvement group (class A3) if they exhibited more wisdom, optimism, hope and peaceful disengagement in step 1. In step 2, psychological flexibility emerged as a significant protective variable and psychological inflexibility as a risk factor. Only the wisdom effect appeared to be substantially reduced when controlling for psychological (in)flexibility, although still significant. Psychological flexibility significantly mediated the effects of wisdom (*z* = − 3.71; *p* < 0.001), optimism (*z* = − 1.68; *p* < 0.05), hope (*z* = − 3.42; *p* < 0.001) and peaceful disengagement (*z* = − 3.16; *p* < 0.001). Psychological inflexibility significantly mediated the effects of wisdom (*z* = − 3.02; *p* < 0.01), hope (*z* = − 2.67; *p* < 0.01) and peaceful disengagement (*z* = − 2.61; *p* < 0.01) in the expected direction, but not for optimism (*z* = − 0.84; *p* = 0.2).

## Discussion

One of the main goals of this longitudinal study was to investigate patterns of individual differences in depression and anxiety in the context of an (un)lockdown process, and then to gain insight into the psychological resources that may promote resilience in such contexts. A preliminary step, therefore, was to test for the existence of such differences and the presence of identifiable patterns of resilience. The next step was to investigate the predictors of membership in the different models. We briefly discuss the results of the preliminary stage before turning to the question of underlying psychological resources.

### Most individuals were resilient to (un)lockdown (H1)

The majority of respondents presented resilient trajectories of depression (79.6%) and anxiety (59.0%) during (un)lockdown. This finding is consistent with previous studies on minimal-impact resilience, in that most people respond to PTE with resilience, with a higher mean prevalence across studies for depression (59.1%) than for anxiety (52.7%)^8^.

Respectively, 7.9% and 27.8% of participants exhibited chronic levels of depression and anxiety. In the chronic anxiety group, anxiety tended to decrease with time, with a steeper slope after unlockdown. On the contrary, chronic depression appeared to increase until the end of lockdown and then to decrease. Another group (11.1% of participants) manifested a drastic decrease in depression during the entire (un)lockdown period. Together, these results indicate that lockdown had a substantive impact on anxiety and depression levels in a significant proportion of people, most of them trending back to a baseline asymptomatic level at about the end of the lockdown or some time afterward. In other words, the lockdown might have had a negative impact on people’s mental health, and when people were released from lockdown or anticipated it, they tended to recover. However, in absence of data recorded prior to the lockdown, caution should be exercised before concluding that it was the lockdown itself that had a particular impact on mental health^18^. The first lockdown was not predictable, but the pandemic situation seems to persist and new lockdowns may be imposed. An interesting research approach would be to assess people’s mental health from the time a lockdown is announced until the lockdown has ended.

Interestingly, 1.5% of depression trajectories showed an increase in symptoms throughout the (un)lockdown process, and 13.2% of anxiety trajectories showed a rapid increase peaking just after unlockdown, followed by a rapid decrease. Analyses of daily activities showed that the participants in these two groups were less likely to work outside their home during the lockdown than telework from home. As a result, these individuals may have enjoyed the experience of lockdown, for example, by being free from external duties, so that the return to normal life became a stressful experience. These results confirm our hypothesis of individual differences in responses to unlockdown, some experiencing it as a stressful event and others not.

This leads to a potential limitation of the methodological approach used here. Some authors argued that resilience must be inferred as a function of the interaction between experienced stressors and mental health over time^86^. Under such a view, one might not agree to characterise as ‘resilient’ people who displayed good mental health if they had not experienced lockdown as a stressful event in the first place. In contrast, our approach consisted in uniformly inferring resilience from good mental health, regardless of how individuals initially responded to the potential stressor constituted by the pandemic and lockdown situation. A similar approach was applied in a lockdown context^38^. Our point is to consider that the ability to cope easily with stressful events is an integral part of resilience, which justifies studying the resources underlying this ability and led us to put the following hypothesis to the test. Additionally, the time frame of the study was limited to the first French (un)lockdown. As mentioned in the introduction, only minimal-impact resilience can be studied following acute PTEs—that is, the manner in which individuals tend to see their well-being as not impacted during the process^12^. As the pandemic situation appears to be long-lasting, it would be important to evaluate emergent resilience also^12^, i.e., how individuals reconfigure their habits and well-being in response to a lasting change in their environment.

### Positive psychological resources predict resilient trajectories (H2)

Wisdom, optimism, hope, peaceful disengagement and to a lesser degree self-efficacy consistently predicted membership of the resilient trajectories over the symptomatologic trajectories, even after controlling for demographic variables (see step 1 in Tables 5 and 6). The other psychological resources showed no effects when controlled by other resources. In most cases, wisdom appeared to be the strongest protective variable among the psychological resources and was associated with improved resilience against almost any other symptomatology classes. To our knowledge, no studies have been specifically dedicated to the evaluation of the impact of wisdom on mental health. Available evidence in the literature has shown that wisdom and well-being are positively related, with a stronger relationship when people are facing adversity^43^. The second most powerful protective psychological resource was peaceful disengagement, which is a detached and tranquil attitude toward external accomplishments. It was related to resilience against almost all other symptom categories. However, this variable was associated with a greater decline in well-being over time during lockdown^39^. This suggests that peaceful disengagement may be a good strategy for protecting mental health during (un)lockdown, probably because it helps people to disengage from stressful demands, but this comes at a cost to happiness, perhaps because disengagement reduces the opportunity to enjoy the achievements that could have been attained otherwise. Optimism was consistently associated with increased likelihood of membership in the resilience class over other classes, yet with a lower odds ratio magnitude than wisdom and peaceful disengagement. In particular, the depression with improvement class (D1) was only predicted by optimism. It should be noted that optimism has many denotations. Here, optimism was assessed as a disposition towards positive expectations. It was associated most with fewer depressive episodes, whereas optimism as a sense of invulnerability moderated the effect of life stress on anxiety^87^. Hope was only significantly associated with resilience against the decreasing depression (D4) and the chronic anxiety (A3) trajectory classes. These two classes shared a high initial level of mental illness with a progressive improvement over time. We might assume that hope would be a better predictor of recovery than of resilience, meaning that hope would not protect mental health when the stressful situation has occurred when controlled by other resources, but it does help one to recover more quickly. The last protective resource found was self-efficacy, which significantly protected individuals from experimenting the decreasing depression trajectory (D4). This effect was fully mediated by psychological flexibility.

These findings are consistent with previous research showing that positive mental dispositions are strong predictors of resilient trajectories in the face of PTEs^12^. Interestingly, the positive psychological resources we identified as protective were consistent with regard to their relationships to anxiety and depression, but draw a picture that is somewhat different from previous findings related to well-being during lockdown^39^. In the latter study, emotional well-being was positively predicted by hope, gratitude of being and acceptance; psychological well-being by self-efficacy, personal wisdom and gratitude of being; social well-being only by gratitude toward the world and inner well-being by optimism, gratitude of being and acceptance. However, the earlier study did not attempt to predict resilient trajectories but only the main effects of these resources on well-being intercepts and slopes, as well as their moderation of the effects of perceived health and economic threats. To our knowledge, the present study is the first to jointly test the protective effects of a wide range of psychological resources in response to PTEs with resilient trajectories as an outcome. A nice avenue of research would be to evaluate whether these psychological resources are consistently protective against other PTEs, as well as promoting other mental health or well-being trajectory outcomes. Indeed, we do not claim exhaustivity; some relevant positive psychological resources, such as mindfulness^88^, self-compassion^89^, etc., were not tested in this study.

Besides psychological resources, certain sociodemographic and environmental factors exhibited a significant positive relationship with resilience. Age, relationship quality and ‘not being alone’ were positively associated with resilience to depression, whereas being male, being alone and both environment and relationship quality were positively associated with resilience to anxiety (the strength of the effects varied however across symptomatologic classes). Working outside the home appeared as a risk factor for anxiety and depression trajectories. These results add to the literature, which persistently showed that trajectories are moderated by positive psychological resources, demographic characteristics and environmental factors^8^.

### Psychological flexibility mediates the protective effects of psychological resources (H3)

It has been hypothesized that psychological flexibility—the individual disposition to have the capacity to adapt one’s psychological thoughts and behaviour to the situation—may mediate the protective effects of psychological resources. We have seen that psychological flexibility appeared to be consistently more powerfully related to resilient trajectories than psychological resources (see step 2 in Tables 5 and 6). The same was true for psychological inflexibility in the expected direction, but it was only significantly related to the decreasing depression and increasing anxiety groups (Classes D4 and A3).

All in all, psychological flexibility appeared to be an important mechanism mediating the relation between psychological resources and depression. However, the number of mediated effects depended on the psychological resources and the mental health outcome considered. We speak of total mediation when the respective psychological resource is not significant in step 2, and of partial mediation otherwise (see Tables 5 and 6).

Wisdom effects were always mediated by psychological flexibility, and fully meditated for the depression groups. Consistently, wisdom was the best predictor of psychological flexibility when controlling for all psychological resources (see Tables S1 and S2). Hope and optimism showed a similar pattern when considering the anxiety and depression classes separately. The mediation of psychological flexibility tended to be stronger with trajectories of depression than with anxiety. Optimism was significantly and fully mediated by psychological flexibility for the persistent and decreasing depression classes (D2 and D4). Hope and self-efficacy also appeared to be totally mediated for the decreasing depression class (D4). In contrast, optimism was not mediated for the increasing depression class (D1) and was only weakly and partially mediated for the anxiety symptomatology classes. This finding is in line with a previous study during the pandemic, where both optimism/pessimism and psychological flexibility significantly mediated coronavirus stress effects on a composite factor of psychological problems, including depression and anxiety^64^. Hope was also only weakly and partially mediated with the improved anxiety class (A3). This suggests that psychological flexibility is an important mechanism in the relationship between optimism and hope with depression trajectories, but not so much for anxiety. Finally, peaceful disengagement was always significantly, but not totally and not strongly, mediated by psychological flexibility. Thus, the mediating mechanism explaining class membership may differ from one psychological resource to another. For some resources, such as wisdom, psychological flexibility mediation appears particularly relevant. Central to personal wisdom, as construed here, is the reflective dimension, which is the individual propensity to step back and look at phenomena from different perspectives^71^.

An alternative mechanism between psychological factors and minimal impact resilience, such as the unexplained optimism and hope effects on anxiety trajectories, is the positive appraisal of potential stressors^90^. Accordingly, the positive reappraisal mechanism was found for optimism^91^, hope^92^ and gratitude^93^. Also, mindfulness meditation has been shown to be robustly associated with positive functioning through positive reappraisal^94,95^. The combination of the two mediation mechanisms might explain many of the effects of psychological resources. For example, the protective effects of hope on post-traumatic growth in adolescents following an earthquake were found to be mediated by both acceptance and positive reappraisal^96^. However, we are not aware of a study evaluating jointly the two mechanisms with multiple psychological resources, and more longitudinal studies will be needed to better understand the mechanism of resilience factors.

These results are in line with other Covid-19 and lockdown studies that have shown a predominant mediating role of psychological flexibility for the preservation of mental health^60,97^. The proposed mediation mechanism was very robust, with most protective effects of psychological resources mediated by psychological flexibility. Psychological inflexibility appeared generally less related to mental health trajectories and mediated the effects of psychological resources to a lesser extent than psychological flexibility. Psychological inflexibility and flexibility are mutually related but independent processes^56,98^. According to the Hexaflex model, psychological in/flexibility is respectively underpinned by six interdependent core processes (i.e., 12 dimensions). In this article, the two scales used to assess psychological in/flexibility (i.e., AFQ and AAQ) measured distinct processes: experiential avoidance and cognitive fusion for AFQ, and acceptance and action for AAQ. In the literature, studies showed different implications of these six core dimensions depending on the measure used and the outcomes explored^98^. In addition, these two measures are rarely used simultaneously, which makes it difficult for researchers to distinguish which dimension of in/flexibility influences trajectories of mental health, and which more distal factors (psychological resources in our case) can be involved in this relationship. Our findings are consistent with the distinct role of psychological flexibility and inflexibility and with that of acceptance and action, more than experiential avoidance and cognitive fusion, as protective processes, potentializing the role of the positive psychological resources assessed. Moreover, the majority of our sample followed a resiliency trajectory, suggesting the predominance of a psychological flexibility pattern.

Some researchers have explored the role of psychological in/flexibility on mental health and the factors promoting the different dimensions of psychological in/flexibility. Mindfulness, for example, appears to be one of the resources that can buffer cognitive fusion and promote mental health^99^. Self-compassion also plays a distal role on depressive symptoms through the avoidance process^100^. In the case of the Covid-19 pandemic, a mediating role of psychological flexibility has been found between the disposition to be anxious about one’s health and health outcomes (anxiety and depression)^101^. The measure of each psychological in/flexibility dimension and their respective determinants in explaining mental health trajectories appears to be particularly relevant to discriminate what specific treatments can be proposed according to specific mental health trajectories. A global catastrophic event such as the Covid-19 pandemic highlights the importance of exploring the proximal and distal mechanisms explaining interindividual differences in susceptibility to lockdown situations. Concretely, the study by Kashdan^102^ is an example consistent with the idea that trauma-related distress can generate health benefits (i.e., post-traumatic growth and meaning) only if individuals do not avoid their thoughts, feelings and sensations following the trauma. Consequently, identifying psychological factors that would reduce experiential avoidance could be a promising avenue to design interventions to foster mental health.

Some limitations of the mediation inference remain to be noted. Psychological resources and flexibility were measured simultaneously in the first wave. This design prevented us from inferring causality and might alter the true longitudinal mediation estimates^103^. For a better test of the mediation mechanism, the independent, dependent and mediating variables should all be measured at least three times, allowing for the estimation of the lagged paths. A more general limitation of this study is that our sample was mainly composed of female and French participants, preventing generalisability of the results to the entire population. It would be important to see if the results could be replicated with more representative samples and other cultures as well.

## Conclusion

In this longitudinal study during and after the first French lockdown, we investigated the protective roles of psychological resources and flexibility on mental health trajectories. Results have shown that the majority of people displayed resilient depression and anxiety trajectories. Five psychological resources, namely wisdom, optimism, self-efficacy, hope and peaceful disengagement, predicted membership of the resilient class, and many of these protective effects appeared to be mediated, at least partially, by psychological flexibility. This study showed how important, in a real situation, individuals’ positive psychological dispositions are for the preservation of mental health in lockdown. The results may be relevant to the prevention of mood disorders in such situations. It is our hope that this study has pointed to the necessity of promoting the development of psychological resources in normal times so that mental health can be protected in difficult times to come.

## Supplementary Information

Supplementary Information.

## Data Availability

The datasets generated during and/or analysed during the current study are available in the Open Science Network repository, https://osf.io/q2e6h/.
