# Supplementary material for: Psychological resources and flexibility predict resilient mental health trajectories during the French covid-19 lockdown
Source: Sci Rep. 2022 Jun 23;12:10674. doi: 10.1038/s41598-022-14572-5 (PMC9219392; doi:10.1038/s41598-022-14572-5)
Supplement: Supplementary file 1 — Supplementary Information. [file 41598_2022_14572_MOESM1_ESM.pdf]

Psychological resources and flexibility predict resilient mental health trajectories during the French covid-19 lockdown : Supplementary Figures and Tables

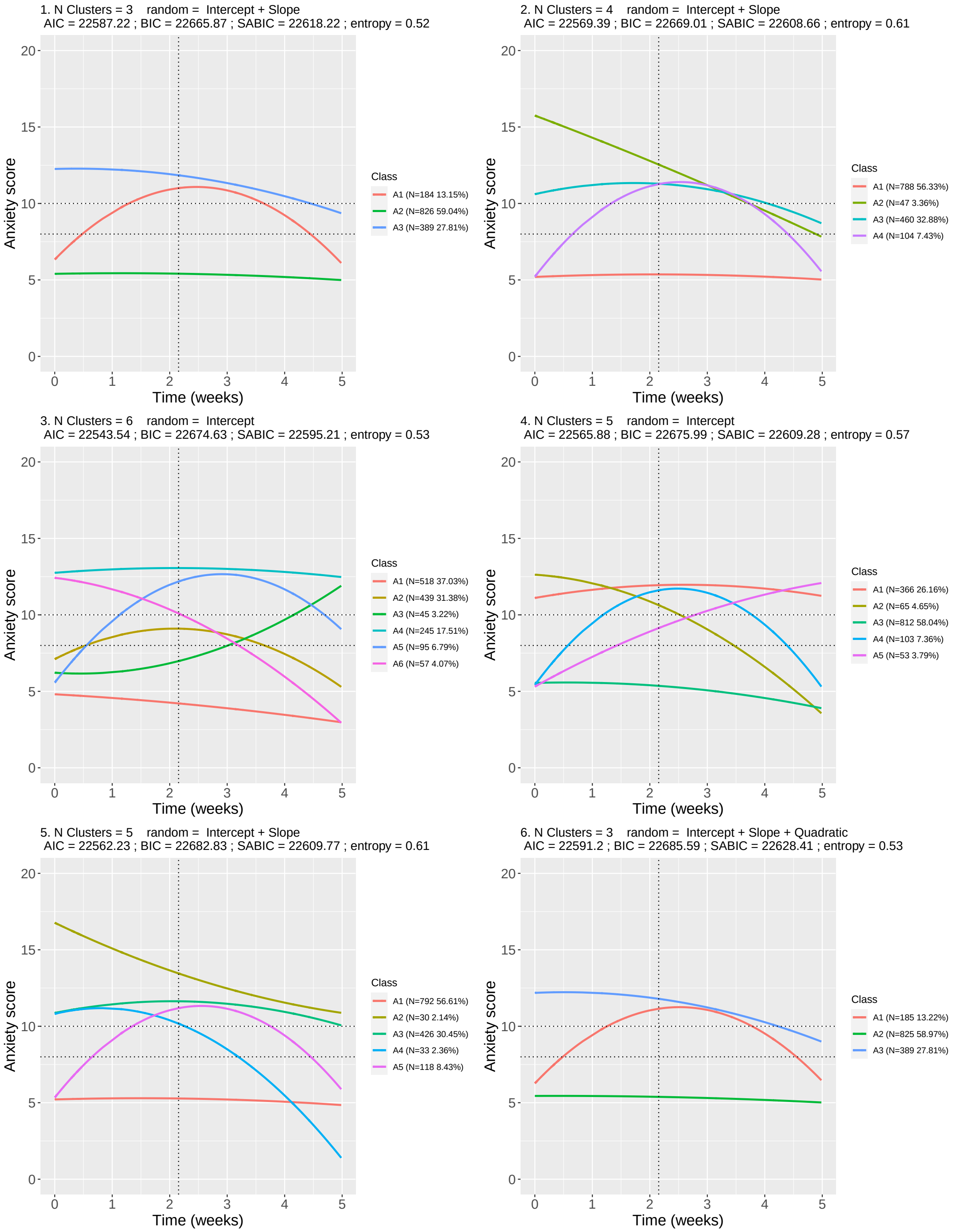

Figure S1. Predicted trajectories from the six best growth mixture model of depression ranked by BIC. Dashed horizontal lines represent symptomatic thresholds for depression (i.e., 8 and 10). The dashed vertical line depicts the moment unlockdown happened.

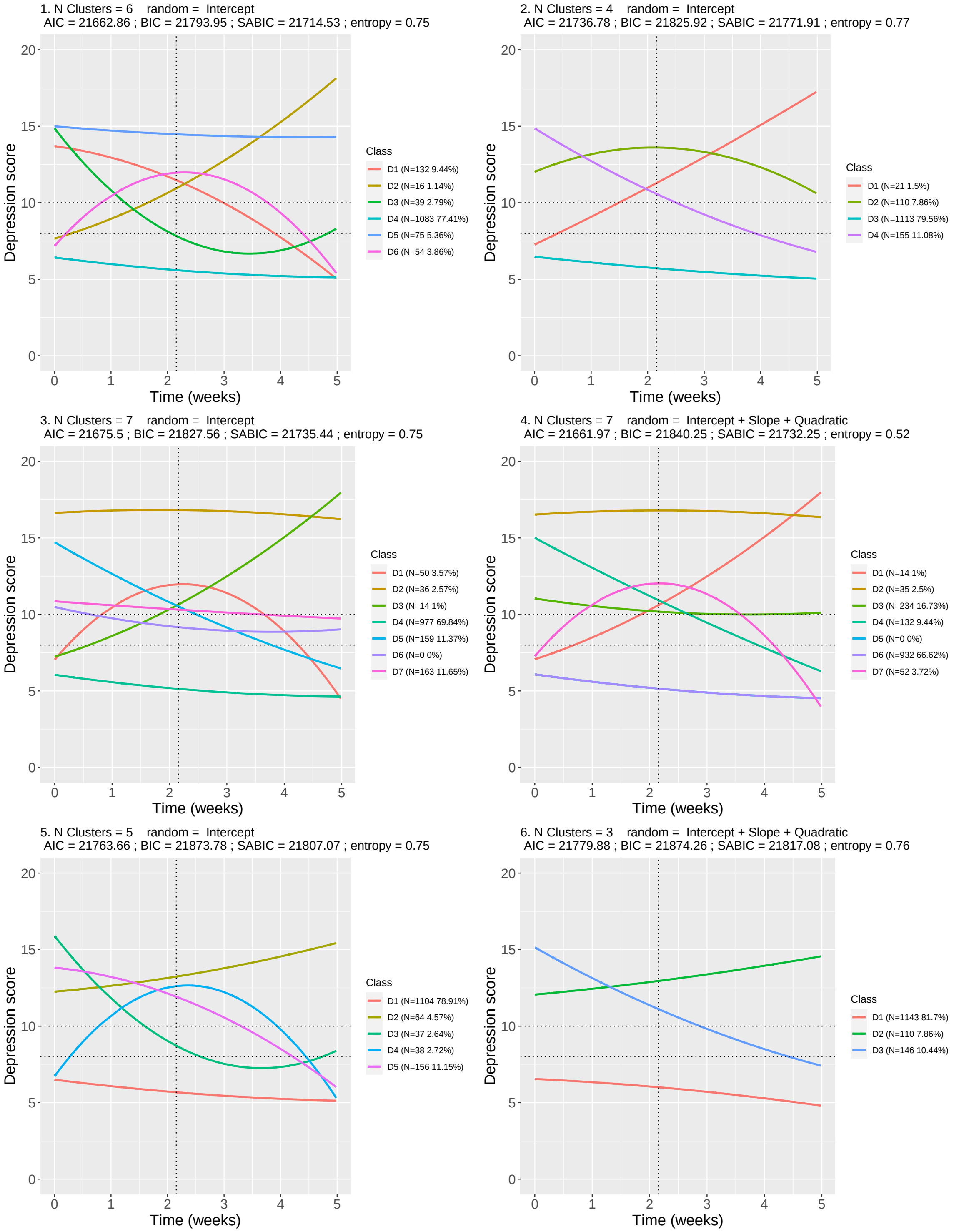

Figure S2. Predicted trajectories from the six best growth mixture model of anxiety ranked by BIC. Dashed horizontal lines represent symptomatic thresholds for anxiety (i.e., 8 and 10). The dashed vertical line depicts the moment of unlockdown.

Table S1

Results of the linear regression model with psychological flexibility (AAQII) being predicted by psychological resources.

Predictor	b	95% CI	$t(1390)$	p
Intercept	-0.34	[-0.65, -0.03]	-2.18	.029
Gratitude-world	0.04	[-0.01, 0.09]	1.62	.105
Self-Transcendence	-0.11	[-0.15, -0.07]	-5.07	$< .001$
Wisdom	0.51	[0.45, 0.58]	15.84	$< .001$
Optimism	0.04	[0.00, 0.08]	1.94	.053
Self-efficacy	0.08	[0.03, 0.13]	3.41	.001
Hope	0.20	[0.15, 0.25]	7.90	$< .001$
Gratitude-being	0.08	[0.04, 0.12]	3.50	$< .001$
Peaceful disengagement	0.13	[0.09, 0.18]	5.80	$< .001$

Table S2

Results of the linear regression model with psychological inflexibility (AFQ) being predicted by psychological resources.

Predictor	b	95% CI	$t(1389)$	p
Intercept	4.94	[4.69, 5.19]	38.77	$< .001$
Gratitude-world	-0.05	[-0.09, -0.01]	-2.28	.023
Self-Transcendence	0.09	[0.06, 0.13]	5.34	$< .001$
Wisdom	-0.47	[-0.52, -0.42]	-17.91	$< .001$
Optimism	-0.02	[-0.05, 0.02]	-0.92	.356
Self-efficacy	-0.01	[-0.04, 0.03]	-0.31	.754
Hope	-0.12	[-0.16, -0.08]	-5.64	$< .001$
Gratitude-being	0.01	[-0.03, 0.04]	0.34	.732
Peaceful disengagement	-0.10	[-0.13, -0.06]	-5.21	$< .001$
